# Abortion patients’ perspectives on enhancing a telemedicine model of post-abortion contraception: a qualitative study

**DOI:** 10.1136/bmjsrh-2024-202428

**Published:** 2024-09-04

**Authors:** Nicola Boydell, Sophie Buijsen, John Joseph Reynolds-Wright, Sharon T Cameron, Jeni Harden

**Affiliations:** 1 Usher Institute, Centre for Biomedicine, Self and Society, College of Medicine and Veterinary Medicine, The University of Edinburgh, Edinburgh, UK; 2 Science, Technology and Innovation Studies, School of Social and Political Science, The University of Edinburgh, Edinburgh, UK; 3 Institute for Repair and Regeneration, Centre for Reproductive Health, The University of Edinburgh, Edinburgh, UK; 4 Chalmers Centre, NHS Lothian, Edinburgh, UK; 5 Usher Institute, Centre for Population Health Sciences, College of Medicine and Veterinary Medicine, The University of Edinburgh, Edinburgh, UK

**Keywords:** Abortion, Therapeutic, abortion, induced, contraception behavior, Counseling, Health Services Research, qualitative research

## Abstract

**ABSTRACT:**

**Background:**

Access to post-abortion contraception (PAC) is critical for reducing unintended pregnancies and supporting reproductive decision-making. Patients often face challenges in identifying, accessing and initiating their preferred contraceptive methods post-abortion. This may be particularly so with telemedicine models of care with absence of in-person appointments, and reduced opportunities to provide some contraceptive methods. This qualitative service evaluation explored patients' perspectives on PAC consultations and decision-making to inform future PAC service models in the era of telemedicine.

**Methods:**

Qualitative interviews with 15 patients who had telemedicine medical abortion at home up to 12 weeks’ gestation. Data were analysed using reflexive thematic analysis.

**Results:**

Contraceptive discussions during pre-abortion consultations were valued for supporting informed choices about future contraceptive use. Decision-making was influenced by previous contraception experiences, emotional state at the time of abortion and concerns about contraceptive ‘failure’. Some preferred non-hormonal methods due to past negative experiences with hormonal contraceptives. However, limited information about 'natural' contraceptive methods and concerns about discussing these with healthcare professionals were described. Barriers to accessing preferred methods, particularly long-acting reversible contraception (LARC), included reduced availability of appointments and caring responsibilities. Fast-tracked appointments for LARC fitting post-abortion were valued. The need for flexible PAC consultations and access after abortion, for example, remote consultations complemented by personalised interactions with sexual and reproductive health experts, was emphasised.

**Conclusion:**

The findings highlight the need for flexible and more accessible PAC service models in the era of telemedicine care to ensure timely access to preferred contraceptive methods.

**WHAT IS ALREADY KNOWN ON THIS TOPIC**

- Patients often face challenges in identifying, accessing and starting their preferred contraceptive method after medical abortion. This may be particularly an issue with telemedicine models of care.

**WHAT THIS STUDY ADDS**

- Supporting patients in making informed choices about post-abortion contraception (PAC) and ensuring timely access to a comprehensive range of contraceptive options is critical.

**HOW THIS STUDY MIGHT AFFECT RESEARCH, PRACTICE OR POLICY**

- With telemedicine-delivered abortion care, there is a need for more adaptable PAC service models to enhance the availability of contraceptive options and facilitate informed decision-making, access and initiation.

## Introduction

 Improving access to post-abortion contraception (PAC) is a central recommendation of United Kingdom (UK) and international policy frameworks and evidence-based guidelines.^1^,^4^ UK guidelines recommend that abortion services offer the complete range of reversible contraceptive options to patients at the time of their abortion.^3 4^ In the context of telemedicine-delivered medical abortion care, this typically equates to the same day as a patient’s telemedicine pre-abortion consultation. Ensuring rapid access to contraception after abortion reduces the likelihood of subsequent unintended conceptions.^3 4^ This underscores the value of contraceptive counselling at the pre-abortion consultation, as it can enhance reproductive autonomy by supporting informed reproductive and contraceptive decision-making and help to prevent future unintended conceptions.^4^,^7^

Many patients encounter challenges in identifying, accessing and initiating their preferred contraceptive method after abortion.^8 9^ Previous research has highlighted the complexity patients (see online supplemental file 1) experience in navigating and making decisions about contraception during abortion treatment, including identifying referrals and access, processing a high volume of information, and managing competing emotional, physical and logistical priorities .^8^,^10^ Consequently, contraceptive counselling at the time of abortion can be challenging, potentially raising tensions for both patients and providers.^6 9 10^ Existing research highlights the importance of providing contraceptive counselling in a non-judgmental and person-centred way to avoid the potential for coercion, whether implicit or explicit.^9^,^12^

Over the past three decades, the landscape of abortion care has undergone significant change, driven in part by advancements in medical abortion.^13^,^16^ Since the COVID-19 pandemic, the delivery of early medical abortion throughout Great Britain has largely transitioned to telemedicine.^17^ While telemedicine consultations offer advantages such as enhanced flexibility, accessibility and privacy,^18^ as well as presenting opportunities for tailored discussions on PAC,^19 20^ they also present limitations.^19 21 22^ Although patients can readily obtain short-acting contraception (eg, progestogen-only pill) in abortion medication packs with this model of care, evidence suggests that accessing long-acting reversible contraception (LARC) is more challenging.^23 24^ This can be exacerbated by the need for extra in-person appointments and reduced availability of these effective contraceptive methods in primary care settings.^23 25^ Furthermore, accessing other short-acting contraceptives, such as the combined oral contraceptive pill, may present additional challenges like blood pressure monitoring,^26^ while access to the medium-acting, self-injectable contraceptives may present the challenge of providing self-injection training.

Given these changes in service delivery, further research is needed to understand patients' experiences with contraceptive consultations at the time of telemedicine abortion, and to explore new models of PAC counselling and provision.^17 25^ This article presents findings from a qualitative service evaluation study that explored patients' perspectives on contraceptive counselling and decision-making during telemedicine-delivered medical abortion. The qualitative evaluation addressed three questions: (1) What were patients' experiences with contraceptive counselling during abortion care? (2) What barriers did patients encounter in accessing their preferred contraceptive method? (3) What elements of PAC services do patients value for future service models?

## Methods

This qualitative evaluation took place between May and September 2022 in NHS Lothian, the exclusive provider of abortion care in Edinburgh and the surrounding region, offering approximately 3000 abortions annually. As is typical within the National Health Service (NHS), all aspects of abortion care, including PAC, are provided to patients free of charge. For patients accessing telemedicine abortion, standard care includes an option for discussion of contraception during a pre-abortion telephone consultation with an offer to provide the chosen contraception.^19^ Immediate short- and medium-acting methods can be dispensed, rapid access appointments for LARC offered, and 'bridging' methods provided until LARC can be initiated, if preferred.

We used purposive sampling to select participants from the service. During the recruitment period, in addition to standard care, some patients were offered an enhanced PAC service, including an additional telephone follow-up contraceptive consultation with a nurse.^27^ Our sample included both those who did and did not participate in the pilot PAC consultations. Inclusion criteria included individuals obtaining early medical abortion under Ground C of the 1967 Abortion Act (excluding medical conditions or fetal abnormality), aged 18 years or over and proficient in speaking, reading and understanding English. The principle of information power^28^ guided decisions on sample size, and sample characteristics were reviewed during data collection to ensure a diverse range of experiences were captured.

To recruit, participant information sheets with study contact details were provided to all patients having a medical abortion at home. Patients who used the pilot PAC service were made a further offer of the qualitative study by the nurse consulting with them. A researcher called approximately 3 weeks later to arrange an interview, if agreed.

Individual semi-structured interviews, following a topic guide (online supplemental file 2), explored contraceptive counselling during abortion care, pre- and post-abortion contraceptive methods, factors influencing method choices, barriers to accessing contraception, and perspectives on future PAC service models. Interviews were conducted by social scientists (SB and NB), both cisgender women with experience in sexual and reproductive health (SRH) research. Interviews took place on Microsoft Teams or by telephone and lasted an average of 50 min. They were recorded and then transcribed by a specialist company. Informed consent was obtained verbally, and participants received a £15 voucher in appreciation of their time.

Qualitative data analysis was conducted by NB, SB and JH, following principles of reflexive thematic analysis,^29^ and using NVIVO^30^ for data management. Analysis was iterative and involved repeated readings and comparisons of interview transcripts, which informed the development of a coding framework applied to the entire dataset. During analysis, we drew on sensitising concepts from research literature on PAC and unanticipated issues that emerged during interviews. To ensure analytic rigour, the wider study team engaged in collaborative discussions, drawing on their diverse disciplinary perspectives to interpret the data and develop findings.

The evaluation was reviewed and approved by the NHS Lothian SRH Quality Improvement Team and Edinburgh Medical School Research Ethics Committee (Ref.: 22-EMREC-008).

### Patient and public involvement

Patients and the public were not involved in the planning of this study; however, the interview guide was refined based on participant feedback.

## Results

Of the 54 potential participants who agreed to be contacted, the final sample comprised 15 patients who accessed post-abortion contraceptive counselling following medical abortion treatment. Participant characteristics are outlined in table 1. We present thematic areas from our qualitative analysis of patients' accounts: (1) considering temporal dimensions of contraceptive counselling; (2) balancing interconnected factors in contraceptive decision-making; (3) situating approaches to contraceptive decision support; and (4) navigating access to post-abortion contraception. Illustrative quotes are presented in tables2  3.

**Table 1:** Characteristics of study participants (N=15)

Characteristic	Total (n)
Gender	
Female	14
Non-binary	1
Age (years)	
18–25	4
26–32	7
33–39	4
Highest education level attained	
Secondary school	1
College of further education	2
University	12
Employment status	
Employed	10
Student	2
Unemployed	2
Full-time parent	1
Ethnicity	
White British/Scottish/English/Northern Irish	10
White Other	3
British Asian	1
Prefer not to answer	1
Relationship status	
Single	4
In relationship (not cohabiting)	4
Cohabiting/married	7
Method of abortion	
Medical abortion at home <11 weeks+6 days’ gestation	14
Medical abortion in hospital clinic <11 weeks+6 days’ gestation	1
Previous pregnancy	
Yes	7
No	8
Previous abortion	
Yes	4
No	11
Contraceptive use immediately prior to abortion	
None	4
Condoms	6
Contraceptive pill	2
Natural family planning methods	4
Enhanced NHS Lothian post-abortion contraceptive service	
Offered	13
Accepted	5

Total numbers for ‘Contraceptive use immediately prior to abortion’ exceed sample size (N=15) because some participants reported use of more than one method.

**Table 2:** Illustrative quotes: considering temporal dimensions of contraceptive counselling and balancing interconnected factors in contraceptive decision-making

Theme	Quote #	Illustrative quotes
Considering temporal dimensions of contraceptive counselling	1	*“Cause definitely in more situations than not, it’s probably a very good thing to already get immediately thinking about.”* [P013; age 18–25 years]
	2	*“I was more focused on getting a date and planning for the termination than I was about going back on contraception.”* [P008; age 26–32 years]
	3	“[…] *it would be handy to have a follow-up call, like maybe now to talk again about the options. Because I think I’d be more in the frame of mind to ask questions and actually get something booked in.” *[P011; age 26–32 years]
	4	*“I think what one thing that’s important is just making sure people don't fall between the cracks of, like this… having this like, where they have a gap between abortion and the beginning of contraception. I think that’s quite important that they try and, you know, crank that up when necessary.”* [P001; age 26–32 years]
	5	*“I think that’s the difficulty of having the talk about contraception while you're planning the abortion is that I've said yes to something and they've given me a year’s worth supply of medicine and I feel like I can't go back down. I'm sure I can. But there’s part of me that feels like I can't go back now and be like, actually, I'm not happy with this.”* [P002; age 26–32 years]
Balancing interconnected factors in contraceptive decision-making	6	*“I felt like the choices I was making were my choices. I didn’t feel like I was trying to meet anyone’s expectations.”* [P006; age 33–39 years]
	7	*“Well, they are quite…what’s that word? They really are wanting you to get the coil *[IUD] *and it, kind of, to me did make sense as well because she said that’s the most effective method.”* [P007; age 26–32 years]
	8	*“I did think I'd have to get something more permanent put in again, just to kind of avoid this from happening again.*” [P001; age 26–32 years]
	*9*	*“I think when you mention natural tracking, they just think like the old rhythm method type thing… I think a lot more people are going hormone-free with things like this now just because of a lot of side effects obviously. But I think there should be a bit more knowledge about things like fertility tracking, and hormones.” *[P010; age 26–32 years]

IUD, intrauterine device.

**Table 3:** Illustrative quotes: situating approaches to contraceptive decision support and navigating access to post-abortion contraception

Theme	Quote #	Illustrative quote
Situating approaches to contraceptive decision support	10	*“It was just quite nice, being able to read…the pros and cons of each one, the implications, specifically how certain ones get fitted. And even their little videos were quite nice… It was like a visual thing, was quite nice.” *[P003; age 18–25 years]
	11	*“I felt like I needed that call to discuss other options. And so, I actually was quite grateful that it was going to happen.”* [P005; age 26–32 years]
	12	*“And sometimes doing, especially contraception face to face, kind of makes you think about when you're a teenager, and you're kind of having your first appointment about the pill at the doctor, and it feels a bit, maybe a bit preachy…But because it was over the phone, it wasn’t so much.”* [P010; age 26–32 years]
	13	*“I've went back in, and I've spoken to someone face to face about contraception, and I think that’s probably why I decided to take a pill. Because I don't know, it’s just, it’s easier to speak to people face to face, and in a room about something.”* [P004; age 26–32 years]
Navigating access to post-abortion contraception	14	*“It was an awful shame that I couldn’t have access to the coil *[IUD] *at my GP service…and it was something silly like, I was logging on at midnight to get an appointment, to get into the *[SRH centre] *to get the coil fitted.”* [P004; age 26–32 years]
	15	*“And also, she said that she could, kind of, fast track me if I wanted a coil or something, which was useful information because it’s literally impossible to get an appointment with your doctor at the moment. So that was useful.”* [P011; age 26–32 years]
	16	*“I don't know if I would have got it *[contraception] *as quickly if the text hadn’t come. So maybe that was kind of perfect timing for the text to come, because I probably would have put it off a bit longer.”* [P010; age 26–32 years]
	17	*“Rather than being directed back to my GP, particularly as a twenty something year old that doesn’t really see a GP much, often at all* […] *It feels better to continue on with the people that are already, I’ve discussed like having an abortion with, and I’ve discussed the kind of reproductive health with them already.” *[P013; age 18–25 years]
	*18*	*“And also, GPs are general…they're general practitioners. When it comes to contraception, being able to talk to people who are very knowledgeable on it is, you…yeah, I feel more confident in the decision that I'm making.” *[P008; age 26–32 years]

GP, general practitioner; IUD, intrauterine device; SRH, sexual and reproductive healthcare.

### Considering temporal dimensions of contraceptive counselling

Participants acknowledged the value of discussing contraception during the pre-abortion consultation. Although some had not expected to discuss contraception at that time, it was generally understood as an appropriate point to consider future contraceptive options (Quote #1).

However, some participants noted that their emotional state influenced their level of engagement about ongoing contraception. They emphasised that their primary focus was on abortion treatment rather than PAC decisions (Quote #2). This led some to suggest that they would be more open to discussing contraception after, rather than before, the abortion (Quote #3). Nevertheless, some noted that there may only be one consultation, and if contraception is not discussed at that point, there was a risk that some people may ‘fall between the cracks’ (Quote #4).

Although there was general agreement about the value of discussing contraception pre-abortion, some participants described feeling that they *ought to*, rather than wanted to, discuss contraception *because* they were presenting for abortion (Quote #5).

### Balancing interconnected factors in contraceptive decision-making

Our analysis suggests that, in general, participants did not feel pressure to choose or initiate a specific contraceptive method (Quote #6). However, some perceived an implicit preference for LARC methods among healthcare professionals (HCPs) (Quote #7). Concerns about 'contraceptive failure' motivated some participants to seek different contraceptive methods, including LARC (Quote #8).

Previous negative experiences with hormonal contraceptives, including mood changes, anxiety and reduced libido, influenced contraceptive decision-making, with some participants articulating a preference for non-hormonal methods. Those who had used or considered using 'natural' contraception methods, such as fertility awareness and fertility tracking mobile health applications, described challenges in discussing these options with HCPs due to perceived stigma, doubts expressed about their effectiveness (interpreted as delegitimisation), and an apparent lack of knowledge among HCPs about these methods (Quote #9).

### Situating approaches to contraceptive decision support

Expressing a desire for support in identifying the 'best option' for them, participants valued decision aids and tools, including online contraceptive decision-making tools that synthesise and personalise information. Videos explaining the pros and cons of contraceptive methods were seen as useful and visually engaging (Quote #10). Nevertheless, participants noted the benefit of opportunities for personalised interaction and information from supportive, ‘expert’ HCPs to identify preferences, address concerns about autonomy and control over contraception, and provide further guidance where needed (Quote #11). Several patients expressed a preference for receiving expert advice via telephone due to its convenience and because they felt less judged (Quote #12). However, others, especially those who had experienced a problem related to the medical abortion and therefore had to attend a clinic, highlighted the advantages of receiving contraceptive counselling in person (Quote #13).

### Navigating access to post-abortion contraception

The reduced availability of contraceptive consultations, both within primary care and specialist SRH services, was emphasised by participants, widely attributed to the lasting impact of the COVID-19 pandemic on UK health services. Barriers to accessing contraception were particularly evident in the accounts of those attempting to access LARC methods, such as intrauterine devices (IUDs), prior to the current pregnancy (Quote #14). In contrast, some participants reported finding it ‘easier’ or being ‘fast-tracked’ for an appointment to fit LARC following abortion (Quote #15).

Participants who accepted the offer of receiving text messages offering a contraceptive consultation with a nurse, as part of the pilot programme for enhanced PAC consultations, emphasised the benefits. These messages served as reminders to further consider and access contraception (Quote #16). Continuity of care for contraception from the abortion service was highly valued and identified as a facilitator for contraceptive initiation. Maintaining a relationship with the same HCP was seen as offering benefits, including knowledge of their abortion, contributing to a sense of provider trust and personalised support (Quote #17). In addition, some preferred to remain under the care of community-based SRH services because they were viewed as ‘experts’ in contraception and adept at discussing sensitive issues around reproductive health. General practitioners were widely perceived as less experienced in contraceptive counselling and LARC fitting (Quote #18).

## Discussion

This study provides insights into abortion patients’ experiences of contraceptive decision-making and contraceptive counselling. Our key findings indicate the importance of integrating different approaches to contraceptive decision-making support and challenges in navigating access to PAC, highlighting the diverse needs and preferences of patients for flexible and accessible models of PAC provision.

Participants understood contraceptive counselling as an appropriate aspect of abortion care whether at the time of the pre-abortion assessment consultation or after an interval, enabling informed choices about future contraceptive use. The introduction of telemedicine abortion has increased the volume of information provided remotely, with patients reporting finding it challenging to focus beyond their immediate abortion treatment.^31^ Therefore, providers of telemedicine abortion could consider offering follow-up contraceptive counselling for those who prefer this.^31^

Openness to contraceptive counselling varied among participants and was shaped by previous contraceptive experiences, personal perspectives and abortion experiences, illustrating the recursive nature of decision-making in this context.^68^,^10 32 33^ Concerns about ‘contraceptive failure’ and a desire to avoid another abortion influenced some participants’ decisions to choose long-acting methods like IUDs. This finding is consistent with qualitative studies from Scotland^10^ and Sweden,^9^ where patients experiencing ‘contraceptive failure’ often sought more effective methods after weighing up the perceived advantages.

Not all patients opted to change their contraceptive method post-abortion. Some reported a preference for 'natural' contraception methods, such as fertility tracking apps, both before and after the abortion. This finding echoes research on women’s perceptions of hormonal contraception, including broader concerns about physical side effects (and delegitimisation of these), mental health, sexual function or satisfaction, menstrual changes, distrust of synthetic hormones and concerns about future fertility.^9 32 34^ Some participants described being uncomfortable discussing ‘natural’ methods with HCPs due to perceived knowledge gaps of HCPs on these methods, and stigmatisation of these methods. This aligns with a recent review on fertility awareness-based methods (FABMs), highlighting challenges in accessing accurate information due to the proliferation of period tracking and fertility applications, and insufficient evidence on their effectiveness.^34^ Concerns among providers about effectiveness, and the scarcity of formal training on FABMs, may hinder discussions with patients.^34^,^36^ Further research into concerns about hormonal contraception and patients' perspectives on discussing these with HCPs could offer insights into approaches to supporting contraceptive decision-making.^33^

Barriers to accessing preferred contraceptive methods, especially LARC methods such as IUDs, were identified. Although the COVID-19 pandemic may have exacerbated these issues, difficult access to contraception has been a longstanding issue in the UK.^10^ Timely access to preferred methods is critical, particularly for those facing additional obstacles to making and attending appointments.^22 37^

The importance of convenience, flexibility and accessibility in future PAC service models was emphasised by participants. Remote delivery of contraceptive counselling, supported by decision aids, was acceptable, with the option for in-person consultations when needed, and pre-abortion consultations, highlighting the need for flexibility in timing. This suggests the potential value in offering a separate pre-abortion telephone contraceptive consultation, as suggested by earlier studies.^31^

Specialist SRH services for contraception were often preferred by participants, who perceived HCPs in these settings as more knowledgeable and experienced in counselling and fitting, aligning with past research.^10^ Participants appreciated support during pre-abortion contraceptive consultations, which allowed them to explore contraceptive options and make decisions best suited to their needs. Participants valued a supportive and non-judgmental atmosphere during these discussions because it fostered open dialogue, reduced anxiety and allayed concerns about feeling pressured or coerced into specific contraceptive choices. This is consistent with existing literature that highlights the significance of relational contraceptive counselling and trust, as a lack of trust can negatively impact future contraceptive use.^9 10 32^

Fast-tracked appointments for LARC fitting and same-day appointments for rapid initiation of other methods were viewed by participants as supporting timely access to contraception. This is particularly important given the challenges for patients in accessing contraception in primary care, specialist SRH services, and at the time of abortion, as highlighted by research in the UK during and since the pandemic.^23 25 37^ As such, our findings emphasise the need for flexible post-abortion service models within telemedicine-delivered abortion care to ensure timely access to a range of contraceptive options.

### Strengths and limitations

This study provides rich qualitative insights into patients' experiences with PAC care, which can inform service improvements. However, several limitations should be considered when interpreting the findings. The relatively small sample size recruited from a single region^19^ may limit the applicability of the findings to other contexts. Additionally, the study was conducted in an NHS setting where contraception is provided at no cost, which may affect the transferability of the findings to different service delivery models or geographic contexts where contraception incurs a cost.

## Conclusions

Future research and practice should focus on developing flexible and accessible models of PAC service provision that address patient needs and preferences in the era of telemedicine abortion care. By addressing these findings, services can better meet the needs of patients and improve reproductive health outcomes.

## Supplementary material

10.1136/bmjsrh-2024-202428 online supplemental file 1

10.1136/bmjsrh-2024-202428 online supplemental file 2

## Data Availability

Data are available upon reasonable request.
