# Supplementary material for: Abortion patients’ perspectives on enhancing a telemedicine model of post-abortion contraception: a qualitative study
Source: BMJ Sex Reprod Health. 2024 Sep 4;51(4):e202428. doi: 10.1136/bmjsrh-2024-202428 (PMC12573427; doi:10.1136/bmjsrh-2024-202428)
Supplement: online supplemental file 1 [file bmjsrh-51-4-s001.pdf]

Box 1. Terminology - Abortion patients’ perspectives on enhancing a telemedicine model of post-abortion contraception: a qualitative study

Box 1. Terminology

Patient(s): Throughout this article, the term ‘patient(s)’ is used. Recent research[1] suggests that individuals seeking abortion may prefer the term patient. Patient is also a gender-neutral term referring to individuals receiving medical care. While most individuals receiving abortion care are cisgender women, others also receive abortion care and patient(s) is an accurate, clear, and concise term that encompasses people seeking abortion in clinical contexts.

Early medical abortion: Throughout this paper, the term 'early medical abortion' (EMA) is used to refer to medical abortion treatment up to 11 weeks + 6 days of gestation, in line with World Health Organization recommendations. It is important to note that there are legal variations across Great Britain, with EMA available in a patient’s home up to 11 weeks + 6 days in Scotland, but this is restricted to 9 weeks + 6 days in England and Wales.

Natural Contraception: We acknowledge that the term ‘natural contraception’ is imprecise. In this study, participants commonly associated ‘natural methods’ with fertility awareness methods, such as fertility awareness and fertility tracking mobile health applications. For a discussion of the terminology of natural family planning (NFP) and Fertility Awareness-Based Methods (FABMs), see Duane and colleagues[2].

1. Blaylock R. Patient and Public Involvement (PPI) in abortion research: an exploratory survey. *BMJ Sexual & Reproductive Health* 2023;49(1):69. doi: 10.1136/bmjsrh-2022-201648
2. Duane M, Stanford JB, Porucznik CA, Vigil P. Fertility Awareness-Based Methods for Women's Health and Family Planning. *Frontiers in Medicine* 2022;9 doi: 10.3389/fmed.2022.858977
