# Supplementary material for: Abortion patients’ perspectives on enhancing a telemedicine model of post-abortion contraception: a qualitative study
Source: BMJ Sex Reprod Health. 2024 Sep 4;51(4):e202428. doi: 10.1136/bmjsrh-2024-202428 (PMC12573427; doi:10.1136/bmjsrh-2024-202428)
Supplement: online supplemental file 2 [file bmjsrh-51-4-s002.pdf]

Topic guide - Abortion patients' perspectives on enhancing a telemedicine model of post-abortion contraception: a qualitative study

**Topics**	**Focus/Prompts – indicative questions**
**Introduction**	• Introductions.<br>• Topics and issues to be covered.<br>• Participant's control over the discussion.<br>• Freedom to decline to answer questions.<br>• Withdrawal.<br>• Regular check-ins for participant's comfort.<br>• Confidentiality assurances (anonymity in transcripts and reports).
**Consent**	• Obtain verbal consent for each statement.<br>• Audio record the consent process.
**General background**	**"Can you tell me a little bit about yourself?"**<br>• Use pre-interview questionnaire as prompts.<br>• Discuss the type of abortion consultation (telemedical/telemedicine, telemedicine and face-to-face, or face-to-face only).<br>• Follow-up contraceptive consultation offered and accepted?
**Past experiences of contraception/ contraceptive methods**	**"Before we talk about your experiences of accessing contraception after your abortion, it would be helpful to get some background on your past experiences with using contraception. Can you tell me about that?"**<br>• Past contraceptive use.<br>• Experiences with different methods.<br>• Access and engagement with healthcare professionals (e.g., GP, SRH etc).<br>• Methods tried, likes/dislikes.<br>• Previous planned/unplanned pregnancies (including previous abortion(s)).<br>• Influence of others (partner, friends, family) on contraceptive choices.<br>• Impact of life stage and events on contraceptive decision-making.
**Experience of teleconsultation**	**"Moving now to talk about your experience with [name of service]. Can you tell me a bit about your experience of the consultation?"**<br>• Accessing the service and referral process.<br>• Initial contact with clinic/services.<br>• Feelings about the consultation being by phone.<br>• Choosing the timing of the call (issues around timing, privacy, childcare, work etc.).<br>• Information provided about abortion during the consultation.
**Contraception counselling during the consultation**	**"Can you tell me about the discussion of contraception during the consultation?"**<br>• Discussion with healthcare professional(s) about contraception.<br>• Expectations of contraception discussion/counselling.<br>• Questions about contraception, and if they were answered.<br>• Decision-making about contraception before the consultation.<br>• Resources/support used to support decision-making.<br>• Influence of healthcare professional on decision-making.<br>• Perceived expectations to choose a contraceptive method.<br>• Suitability of the timing for discussing contraception.

Topic guide - Abortion patients' perspectives on enhancing a telemedicine model of post-abortion contraception: a qualitative study

**Immediate access to contraception**	**"Please can you tell me about contraception you were offered at the time of the abortion?**<br>• Contraceptive supplies offered during the consultation.<br>• Ability to obtain the desired contraceptive method.<br>• Contraceptive supplies offered/provided meet needs?
**Post-abortion contraception**	**"Can you tell me about your experiences of using contraception since your abortion?"**<br>• Contraception, if any, used since the abortion.<br>• Consistency/alignment with planned method, post-abortion.<br>• Influences on choice of contraceptive method or decision not to use contraception.
**Access to post-abortion contraception**	**"Can you tell me about any things have made it easier or harder to access and/or use contraception since you had an abortion?"**<br>• Barriers, if any, to accessing preferred contraceptive method.<br>• Factors facilitating access to preferred contraception.
**Experience of post-abortion contraception/ contraceptive services**	**"Thinking back, can you tell me about your overall experience of post-abortion contraception/contraceptive services?"**<br>• What was good/not good about post-abortion contraception/contraceptive services?<br>• Suggestions for changes in post-abortion contraceptive offer.<br>• Desired offers and methods of provision.
**Future services and models of contraceptive provision**	**"Thinking about your experiences, what would an 'ideal' post-abortion contraceptive service look like?"**<br>• Probe preferences for different models of post-abortion contraception (e.g., telephone consultation, online information, pharmacy supplies, LARC fitting clinics, GP consultations, online decision aids).
**Close**	• Thanks, brief summary of interview discussion<br>• Ensure participant has opportunity to add comments/ask questions<br>• Seek feedback on the interview experience
